# Population Screening Using Sewage Reveals Pan-Resistant Bacteria in Hospital and Community Samples

**DOI:** 10.1371/journal.pone.0164873

**Published:** 2016-10-25

**Authors:** Lital Meir-Gruber, Yossi Manor, Shiraz Gefen-Halevi, Musa Y. Hindiyeh, Fernando Mileguir, Roberto Azar, Gill Smollan, Natasha Belausov, Galia Rahav, Ari Shamiss, Ella Mendelson, Nathan Keller

**Affiliations:** 1 Microbiology Laboratory, Chaim Sheba Medical Center, Tel Hashomer, Israel; 2 Central Virology Laboratory, Public Health Services, Ministry of Health, Chaim Sheba Medical Center, Tel Hashomer, Israel; 3 Sackler Faculty of Medicine, Tel Aviv University, Tel Aviv, Israel; 4 Chaim Sheba Medical Center, Tel Hashomer, Israel; 5 Ariel University, Ariel, Israel; 6 Infectious Disease Unit, Chaim Sheba Medical Center, Tel Hashomer, Israel; Amphia Ziekenhuis, NETHERLANDS

## Abstract

The presence of pan-resistant bacteria worldwide possesses a threat to global health. It is difficult to evaluate the extent of carriage of resistant bacteria in the population. Sewage sampling is a possible way to monitor populations. We evaluated the presence of pan-resistant bacteria in Israeli sewage collected from all over Israel, by modifying the pour plate method for heterotrophic plate count technique using commercial selective agar plates. This method enables convenient and fast sewage sampling and detection. We found that sewage in Israel contains multiple pan-resistant bacteria including carbapenemase resistant *Enterobacteriacae* carrying *bla*_KPC_ and *bla*_NDM-1_, MRSA and VRE. *bla*_KPC_ carrying *Klebsiella pneumonia* and *Enterobacter cloacae* were the most common *Enterobacteriacae* drug resistant bacteria found in the sewage locations we sampled. *Klebsiella pneumonia*, *Enterobacter* spp., *Escherichia coli and Citrobacter* spp. were the 4 main CRE isolated from Israeli sewage and also from clinical samples in our clinical microbiology laboratory. Hospitals and Community sewage had similar percentage of positive samplings for *bla*_KPC_ and *bla*_NDM-1_. VRE was found to be more abundant in sewage in Israel than MRSA but there were more locations positive for MRSA and VRE bacteria in Hospital sewage than in the Community. Therefore, our upgrade of the pour plate method for heterotrophic plate count technique using commercial selective agar plates can be a useful tool for routine screening and monitoring of the population for pan-resistant bacteria using sewage.

## Introduction

Antibiotic-resistant bacteria are a growing problem worldwide and thus possess a major threat to global health. Extensive use of antimicrobial agents in hospital settings and animal husbandry, has led to the emergence of pan-resistant "superbugs", which creates serious ecological and epidemiological challenges for humans [1–4].

Among the most common resistant bacteria are the *Carbapenem-Resistant Enterobacteriaceae* (CRE), that have developed resistance to carbapenems (ertapenem, meropenem, imipenem and doripenem), which are most often the last option for therapy. *Klebsiella pneumonia* carbapenemase (*bla*_KPC_) and New-Delhi Metallo-β-lactamase (*bla*_NDM-1_) are among the most important carbapenemases. [5–8].

Methicillin-resistant *Staphylococcus aureus* (MRSA) and vancomycin-resistant *Enterococci* (VRE) represent the biggest therapeutic hurdles among the gram-positive organisms. [1, 9, 10–13].

There is increasing concern about the growing resistance of pathogenic bacteria in the environment. These resistant bacteria are excreted by humans and animals and have been found in different environmental compartments. Most typically, in hospital effluent, municipal sewage, surface, ground and drinking water, as well as sediments and soil. [14].

During 2006 Israeli hospitals faced a clonal outbreak of carbapenem-resistant *Klebsiella pneumoniae* [15]. Since then, several reports have detected CRE's in hospital settings around Israel [16–19]. As Schwaber et al. noted in their report concerning the spread of *bla*_KPC_ pathogens in Israel, *bla*_KPC_ is almost exclusively healthcare acquired with no significant community transmission. Experience with community acquired extended spectrum β-lactamase (ESBL) microorganisms [20–22] and the knowledge that long term care facilities (LTCF's) have become a source of reintroduction of CRE to acute care hospitals [23], the spread of CRE to the community is possible and should be examined.

Since there are no standard methods or commercial selective plates for environmental sewage samples which meet our needs, we developed a method that utilizes clinical selective plates for environmental sewage samples analysis.

Here we demonstrate that the detection of pan-resistant bacteria can be done on environmental samples using commercial selective agar plates in a manner that could be used in routine screening of the population. The identity of the microorganism and the drug resistance mechanism can then be identified by classical bacteriological identification, sensitivity tests and Real Time-Polymerase chain reaction (RT-PCR).

Using this method we sought to evaluate the presence of CRE, MRSA and VRE bacteria in Israeli sewage from hospitals and community collected from all over Israel, representing the population carriage of pan-resistant bacteria and to establish a convenient and fast method for sewage sampling.

## Materials and Methods

### Sampling Site Information

**Fig 1:**
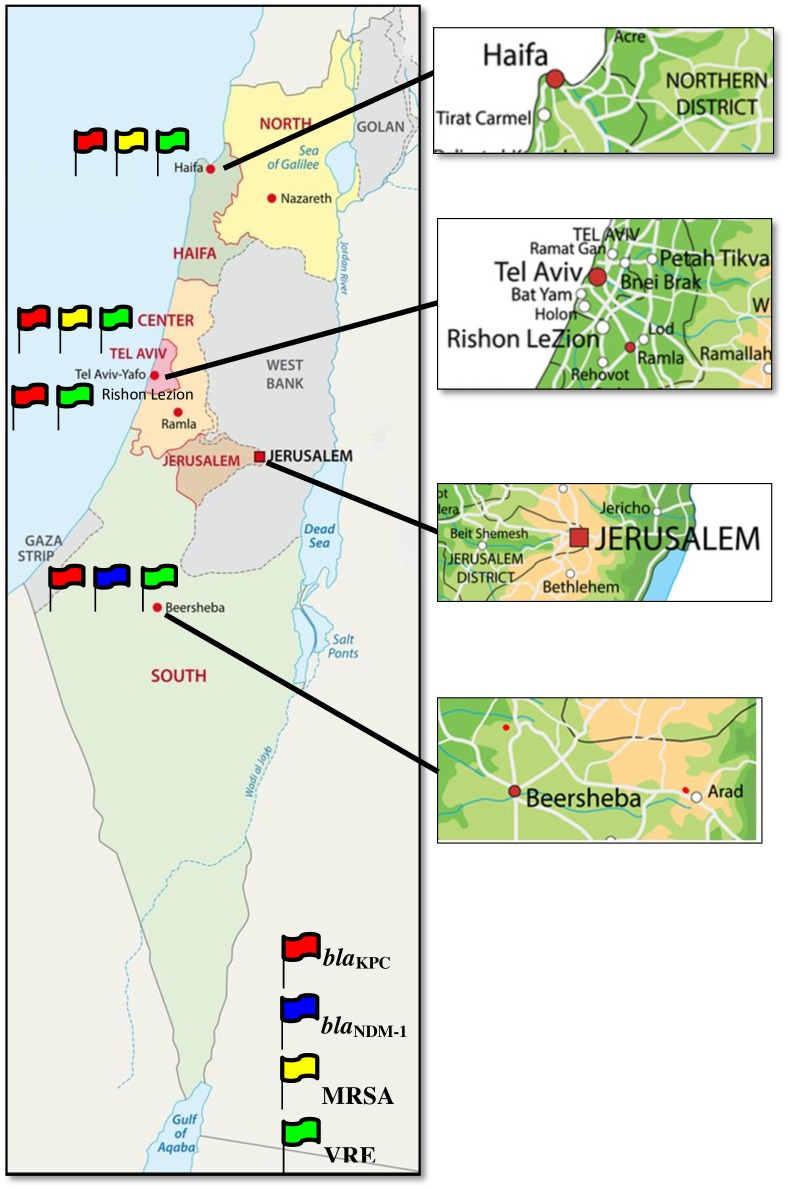
A map of the whole state of Israel illustrating sewage sampling locations. Each location is marked with flags indicating the pan-resistant bacteria found in that location. A magnification of the four main districts is displayed on the right side of the figure. Maps illustrated by "Fotolia by Adobe" (https://www.fotolia.com/).

Two types of sewage sites were analyzed; (1) Sewer systems that drain sewage from the local municipal surroundings and (2) Sewage treatment facility (STF) that drain all the lines of the communities that are included in that line. The sampling locations were divided into four major areas: North, Center, East and South and were categorized as follows: Haifa District: Haifa: Includes the cities Haifa, Nesher, Krayot and Tirat-HaCarmel (STF). Tel Aviv District and Central Region: (1) Tel Aviv: Eight different sampling locations in the city of Tel Aviv-Jaffa (All of the sampling sites in Tel Aviv are sampling sites on sewer systems). (2) ShafDan (Wastewater Treatment Plant of Dan Region): An STF that includes the following cities: Tel Aviv-Jaffa, Ramat-Gan, Bnei-Brak, Giv'atayim, Petah-Tikva, Giv'at-Shmuel, Kiryat-Ono, Tel-HaShomer, Ramat Ef'al, Azur, Bat-Yam, Holon, Rishon-Lezion and Rehovot. Jerusalem City: Three different sampling sites in the city of Jerusalem, which cover the entire city. Due to the unique mountain topography of Jerusalem, each sampling site stands on its own. Sorek and Og are STF's. South District: Three different locations were sampled: Rahat and Kuseife and the biggest city in the south of Israel Be'er-Sheva. Be'er-Sheva and Rahat are STF's (Fig 1 & Table 1) (The Israeli Central Virology laboratory is authorized to sample sewage for routine environmental surveillance as part of the ministry of health of the state of Israel). All sampling locations were also divided by the type of sewage drained; Pipelines that drain sewage from hospitals and/or nursing homes (Hospital) were distinguished from those that drain sewage from the community which do not contain hospitals and/or nursing homes (Community).

**Table 1:** Sampling sites information.

Sampling Sites	Boundaries	Geographical coordinates (Latitude, Longitude)	Population*	Contains Hospital sewage	Resistance genes			
					*bla*_KPC_	*bla*_NDM-1_	MRSA	VRE
					#of positive samples/# of sampling sites			
	***Haifa District***		**462,670**					
**Haifa**	Haifa, Nesher, Krayot and Tirat HaCarmel	32.785965, 35.051865		Yes	2/2	0/2	1/2	1/2
	***Tel Aviv District and Central Region***		**1,620,838**					
**Tel Aviv (TLV)**			**414,565**					
C121 TLV	Kfar Shalem TLV			No	6/8 (75%)	0/8 (0%)	2/8 (25%)	5/8 (63%)
C114 TLV	Kfar Shalem TLV	32.045043, 34.813014		No				
C58 TLV	Yad Eliyahou	32.044491, 34.810208		Yes				
B Line TLV	North TLV	32.061493, 34.787787		No				
A23 Igudan	North TLV &Petah Tikva	32.102600, 34.781872		Yes				
C1 Igudan	South and Central TLV	32.100516, 34.800912		Yes				
Basa Igudan	South TLV & Jaffa	32.100516, 34.800912		No				
Shafdan	**	32.058599, 34.759402		Yes				
	***Jerusalem City (J-m)***	32.100893, 34.901318	**815,308**					
Sorek	West and South J-m			Yes	2/3 (67%)	1/3 (33%)	0/3 (0%)	2/3 (67%)
Og	North and East J-m	31.758565, 35.101516		Yes				
Kidron	South-East J-m	31.800825, 35.258332		No				
	***South District***	31.766390, 35.235947						
Kuseife	Kuseife		17,543	No	2/3 (67%)	1/3 (33%)	0/16 (0%)	3/3 (100%)
Be'er Sheva	Be'er Sheva	31.235874, 35.090786	197,269	Yes				
Rahat	Rahat	31.337678, 34.684747	56,943	No				
**All of Israel**		31.384765, 34.717849			**12/16 (75%)**	**2/16 (13%)**	**3/16 (19%)**	**11/16 (69%)**

### Collection of sewage samples

All sewage samples were collected using automatic composite samplers [24]. The final samples comprised a mixture of 24 or 48 individual samples gathered over a period of 24 hours. Sampling sites were selected following two criteria; (1) Main entrance to a treatment plant: sampling the enteric microbial content of an entire community's sewer system. (2) Specified sites in the sewer system: sampling the enteric microbial content of specific neighborhoods where there were no hospitals or nursing homes.

### Selective overlay plate preparation

Sewage samples were plated according to the pour plate method for heterotrophic plate count technique described in the Standard Methods for the examination of water & waste water [25] with minor modifications. Three different chromogenic media were used as the underlay layer, that are based on the classic chromogenic agar CHROMagar Orientation, enabling easy detection and presumptive identification of bacteria by their specific colored colonies according to their chemical and enzymatic properties; (1) CHROMagarKPC is selective for Gram-Negative Carbapenem-Resistant bacteria, (2) CHROMagarMRSA is selective for *Staphyloccocus aureus* and (3) CHROMagarVRE is selective for VRE (Hy-Labs, Rehovot, Israel).

Briefly, the overlay agar was prepared by diluting 1:1 one of the chromogenic agars with distilled water. The agar was finely chopped and then gently mixed with slightly cooled down boiled distilled water until melted. 1 ml of sewage was added to 6 ml of the pre-melted and diluted chromogenic agar resulting in an overlay with a final dilution of 1:12. The mixture was gently mixed and then poured into 90 mm plates containing the specific CHROMagar growth media and were incubated at 35°C ± 2 for 48 hours.

### Isolation and identification of resistant colonies

From each overlayed CHROMagar plate a certain percentage of suspected to be CRE, MRSA or VRE (according to instructions of manufacturer) were isolated, Gram stained using AerosprayWescor, Slide stainer Cytocentrifuge and observed with a light microscope (Olympus BX43, USA). Identification and susceptibility of CRE and MRSA was performed using the Phoenix^™^ (BD, NJ, USA) and MALDI-TOF MS (microflex LT^™^, Bruker, Germany) systems. Uncertain bacterial identifications were tested by classical/biochemical methods according to the literature [26, 27] and by 16S rRNA sequencing of the first 800bp of the gene (Hy-labs, Israel) for confirmation. Identification and susceptibility of VRE was performed classically according to the literature [26, 27].

### DNA extraction

Automated Nucleic Acid (NA) extractions were carried out from fresh well-isolated colonies by NuclisensEasyMAG (bioMerieux, France) or by EZ1 advanced (Qiagen, Germany) using EZ1^™^ DNA Tissue Kit according to the protocol recommended by the manufacturers. Briefly, from bacterial colonies: a 2.0 McFarland-standard bacterial suspension was prepared from isolated colonies in saline, and bacterial NA was extracted from 200 μl (1.2 X10^8^ CFU) of the suspension. Extracted bacterial NA was eluted from the columns in 100 μl elution buffer and stored at -20°C.

### *bla*_KPC_ and *bla*_NDM-1_ detection by Real RT-PCR

The ABI Prism 7500 sequence detection system (Applied Biosystems, Foster City, CA) was used for the amplification and detection of the *bla*_KPC_ and *bla*_NDM-1_ amplicons by TaqMan technology. For sequences of primers and probes see previously published papers [28, 29].

### Clinical samples

The clinical samples reviewed in this study were blood cultues, general cultues and rectal swabs for screening received by the Clinical Microbiology Laboratory in The Chaim Sheba Medical Center in Israel during 2013. General cultures include synovial, pericardial, pleural and peritoneal fluids, peritoneal dialysate, brain abscess, lung biopsy/tissue, pericardial fluid/tissue, heart valve, bone, joint, internal organs tissues, biopsies, heart valves, electrodes, catheters and wounds. All cultures suspected to be CRE were tested by Modified Hodge test, MBL E-test and by E-tests for imipenem, meropenem and ertapenem (bioMérieux, France) following CLSI guidelines [30].

## Results

### Determination of the different assay's limit of detection (LOD)

In order to determine the limit of detection (LOD) for the overlay method, 1.5X10^8^ CFU/ml *bla*_KPC_ *Klebsiella pneumonia*e, *bla*_NDM-1_ *Escherichia coli*, MRSA or VRE positive bacteria were serially dilluted logarithmically in sewage that was negative for these targets. Diluted bacteria were overlaid (as described above) or classically streaked on selective agar. Plates were incubated for 18–24 hours at 35°C ± 2 and analyzed after 48 hours. We found that the overlay did not cause bacteria death as the LOD was similar (5–10 CFU/ml) between the two methods for the different bacteria.

### Selection of the resistant colonies

All sewage samples that were overlayed on CHROMagar plates exhibited significant growth of colonies. An average of 200–500 colonies of different types of carbapenem resistant bacteria (CRE, *Pseudomans* spp., *Acinetobacter* spp. and *Enterococcus* spp.) grew on each CHROMagarKPC overlayed plate of which an average of 50–100 colonies were big blue or pink, members of the *Enterobacteriacae* family. Of the colonies isolated, 10–15% were tested. An average of 200 colonies of different types of bacteria (*Staphyloccocus aureus*, *Gram positive rods* and *Streptoccocus* spp.) and fungi grew on each CHROMagarMRSA overlayed plate of which an average of 10% were pink colonies that were suspected to be MRSA. Fifty percent of these colonies were tested. An average of 200–300 colonies of different types of bacteria (*Gram negative rods*, *Streptococcus* spp. and *Staphylococcus* spp.) grew on each CHROMagarVRE overlayed plate of which 10–15% were pink colonies suspected to be VRE. Fifty percent of these colonies were tested (Table 2).

**Table 2:** Distribution of the pan-resistant isolates in the different sampling locations in Israel.

	Haifa		Central		Jerusalem		South		Total	
	H	* C	H	C	H	C	H	C	H	C
***bla***_**KPC**_	13/20 (65%)	NA	16/34 (47%)	8/16 (50%)	3/16 (19%)	4/6 (67%)	5/7 (71%)	4/14 (28%)	37/77 (48%)	16/36 (44%)
***bla***_**NDM-1**_	0/20 (0%)	NA	0/34 (0%)	0/16 (0%)	2/16 (13%)	0/6 (0%)	0/7 (0%)	1/14 (7%)	2/77 (3%)	1/36 (3%)
**MRSA**	1/16 (6%)	NA	4/25 (16%)	0/26 (0%)	0/12 (0%)	0/6 (0%)	0/9 (0%)	0/18 (0%)	5/53 (9%)	0/50 (0%)
**VRE**	2/12 (17%)	NA	9/30 (30%)	6/29 (21%)	7/12 (58%)	0/6 (0%)	8/10 (80%)	9/20 (45%)	18/54 (33%)	6/55 (11%)

H, Hospital sewage; C, Community sewage

NA, Not available,

* No sewage samples from Community in Haifa.

### *bla*_KPC_ more common in sewage than *bla*_NDM-1_ in Israel

The presence of pan-resistant bacteria in sewage samples was tested in 16 different sampling locations from April 2012 until November 2013 (Table 1) as described above.

Initially we examined the incidence of *bla*_KPC_ and *bla*_NDM-1_ in sewage samples around Israel. *bla*_KPC_ was detected in most (12/16, 75%) of the locations sampled; Northern Israel, represented by Haifa, had both samplings positive for *bla*_KPC_. In the center of Israel which included 8 locations in Tel-Aviv and the ShafDan-STF, 75% of the locations were positive for *bla*_KPC_. Jerusalem and the South of the country had 67% locations positive for *bla*_KPC_. However, *bla*_NDM-1_ was detected only in 2/16 (13%) of the locations sampled; 1 location in Jerusalem and 1 location in the south (Table 1).

The State of Israel was divided into four main areas for sewage sampling; Haifa district in the north, Central district including Tel-Aviv and the Shafdan-STF, Jerusalem City in the East and the South district. Each district was divided to sub-districts. The boundaries and population size are mentioned for each sampling site. The number and percentage of positive samples for pan-resistant bacteria; CRE (*bla*_KPC_ and *bla*_NDM-1_), MRSA and VRE out of the number of samplings analyzed is indicated.

All geographical coordinates were done using "Google maps" (https://www.google.co.il/maps).

# Haifa sampled twice in the same location therefore no percentage is presented.

* According to Central Bureau of Statistics (CBS) Israel 2012.

** Tel Aviv-Jaffa, Ramat-Gan, Bnei-Brak, Giv'atayim, Petah-Tikva, Giv'at-Shmuel, Kiryat-Ono, Tel-HaShomer, Ramat Ef'al, Azur, Bat-Yam, Holon, Rishon- Lezion and Rehovot

### The incidence of *bla*_KPC_ and *bla*_NDM-1_ in sewage from Hospitals and the Community

**Fig 2:**
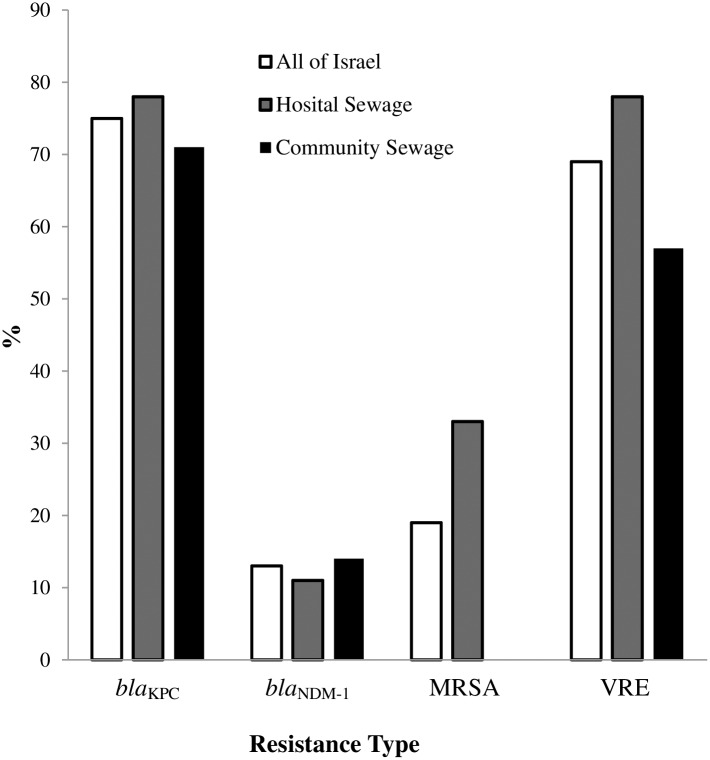
The percentage of pan-resistant bacteria in Israeli sewage containing Hospital compared to Community sewage. No significance was found in the presence of *bla*_KPC_, *bla*_NDM-1_, MRSA and VRE between Hospital and Community sewage (*p* = 0.7533, 0.8588, 0.1143 and 0.2989, respectively).

We compared the percentage of positive sewage samples for *bla*_KPC_ and *bla*_NDM-1_ in sewage that contains hospitals and nursing homes (Hospital) to sewage that does not contain hospital and/or nursing homes (Community). We found no significant differences in the percentage of positive samples for *bla*_KPC_ and *bla*_NDM**-**1_ between Hospital and Community sewage (78% and 63% for *bla*_KPC_ and 11% and 13% for *bla*_NDM-1_) (Fig 2).

Among the locations that do contain Hospital sewage, Jerusalem has the lowest percentage of *bla*_KPC_ positive isolates (19%) compared to the rest of the country (47–71%) but has the most *bla*_NDM-1_ positive samples (13%) compared to the rest of the country (0%) (Table 2).

### The distribution of CRE bacteria in Israeli sewage and clinical samples isolates

Using the selective overlay plate technique enabled us to isolate colonies and to study them. Therefore, we isolated 112 CRE colonies from the different sampling locations. Fifty two out of the 112 (46%) CRE colonies isolated around Israel were *bla*_KPC_ positive by RT-PCR. All *bla*_KPC_ positive bacteria were further analyzed and the distribution of these isolates by genera is shown in Table 3; *Citrobacter braakii* (3), *Citrobacter freundii* (4), *Enterobacter asburiae* (1), *Enterobacter cloacae* (14), *Escherichia coli* (7), *Klebsiella oxytoca* (4*)* and *Klebsiella pneumoniae* (19). Only 3 carbapenem-resistant colonies out of the 112 (3%) were positive for *bla*_NDM-1_ by RT-PCR. Final bacteria identification revealed that 2 isolates were of *Escherichia coli* and 1 was *Klebsiella pneumoniae* (Table 3).

**Table 3:** The number of bacterial isolates carrying the resistance genes *bla*_KPC_ and *bla*_NDM-1_ found in different districts in Israel.

Resistance gene	Bacteria species	District				
		Haifa	Central	Jerusalem	South	Total
***bla***_***KPC***_						
	• *Citrobacter braakii*		2	1		3
	• *Citrobacter freundii*	2	2			4
	• *Enterobacter asburiae*		1			1
	• *Enterobacter cloacae*	3	6	3	2	14
	• *Escherichia coli*	2	5			7
	• *Klebsiella oxytoca*	1	2	1		4
	• *Klebsiella pneumoniae*	5	5	2	7	19
**Total *bla***_***KPC***_		**13**	**24**	**7**	**9**	**52**
***bla***_***NDM-1***_						
	• *Escherichia coli*			2		2
	• *Klebsiella pneumoniae*				1	1
**Total *bla***_***NDM-1***_				**2**	**1**	**3**

**Fig 3:**
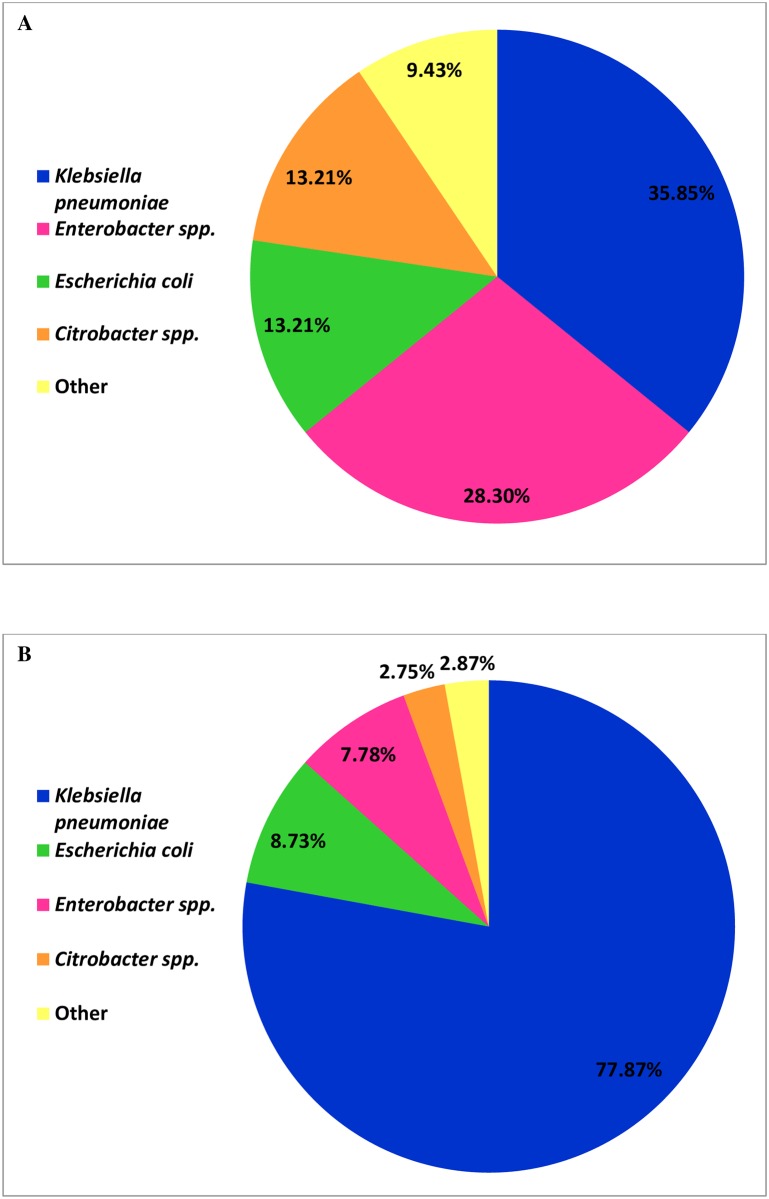
Distribution of *bla*_KPC_ carrying *Enterobacteriacae* in sewage compared to clinical isolates. (A) *bla*_KPC_ carrying *Enterobacteriacae* found in sewage compared to (B) *bla*_KPC_ carrying *Enterobacteriacae* isolates collected in the Microbiology Lab at the Sheba Medical center in 2013. Multiple samples within one patient were excluded.

*bla*_KPC_ carrying *Klebsiella pneumoniae* is also the most common CRE isolated from clinical samples in our clinical microbiology lab. The second, third and fourth most isolated CRE bacteria in our lab are *E*.*coli*, *Enterobacter* spp. and *Citrobacter* spp., respectively. These bacterial species were also the main species found in the sewage (Fig 3B).

### VRE is more abundant in sewage in Israel than MRSA

Overall there were many more positive locations for VRE than for MRSA (69% and 19%, respectively) (Table 1). When examining the differences between Hospital and Community sewage we found more locations positive for MRSA and VRE bacteria in Hospital sewage than in the Community (Table 2). No district had MRSA positive samplings in the Community. Jerusalem and the South district had no samplings positive for MRSA even in Hospital sewage. VRE was positive at all districts from Hospital and Community sewage except from Community sewage in Jerusalem (Table 2).

## Discussion

Since there were no commercial methods for environmental sewage sampling using selective plates to meet our needs, we developed a method that combines the selective ability of the clinical selective plates with the environmental sewage sampling. We found that this method has an average LOD of 5–10 CFU/mL and thus can be easily used by laboratories for sewage screening. We have shown that sewage in Israel contains multiple MDR bacteria including CRE carrying *bla*_KPC_ and *bla*_NDM-1_, MRSA and VRE.

It is most interesting that while we found in our work that the most abundant CRE in sewage in Israel was *bla*_KPC_ carrying *Klebsiella pneumonia* followed by *Enterobacter cloacae*. (Table 3), in a previous publication by Xinzhuo Zhang et al. *bla*_KPC-2_ positive *Citrobacter freundii* and *Enterobacter cloacae* were the most abundant CRE found in hospital sewage in China [31]. The divercification pattern of pan-resistant bacteria in sewage could imply the carrier rate of those organisms in the population. Our finding that *bla*_KPC_ carrying *Klebsiella pneumoniae* followed by *Enterobacter cloacae* and *Escherichia coli* are the most abundant CRE in Israeli sewage correlates with our additional findings that *bla*_KPC_ carrying *Klebsiella pneumoniae* followed by *Escherichia coli* and *Enterobacter* spp. are the most common CRE bacteria found in clinical samples as blood and general cultures and in routine rectal swabs screening in our hospital. This finding also correlates with the fact that *bla*_KPC_ carrying *Klebsiella pneumoniae* was the cause of an outbreak in Israeli hospitals in 2006 and has become a continuous struggle to control it since [16, 17, 32]. *bla*_NDM-1_ carrying bacteria which have also been reported recently in Israeli hospitals [18, 33, 34] were also present in Israeli sewage, though to a much lesser extent (13%) than *bla*_KPC_ (75%) (Tables 1 and 3). Our results show that this method can be a useful tool in screening and maybe predicting bacterial outbreaks in Hospitals and the Community. It is important to emphasize that, though the community is not supposed to be exposed to sewage, once in a while there is an intentional or not flowing of sewage to the ocean or leakage of sewage pipelines which exposes the community to unwanted microorganisms including pan-resistant bacteria. Therefore, we cannot be indifferent towards the microorganism content of sewage as pan-resistant bacteria such as CRE may cause serious infections, especially bacteremia and urosepsis and carries a high associated mortality rate [35–39]. Our study also shows that generally Hospital and Community sewage contain similar types of pan-resistant bacteria in Israel (Fig 2 & Table 2). This is surprising as we would expect to find less resistant bacteria in the community. Therefore, it is not clear if the presence of pan-resistant bacteria in the community is due to person to person transmission having hospitals a significant source of these bacteria or due to other environmental factors such as water or food consumption such as intensively farmed chicken carrying VRE [40] but it emphasizes that those bacteria are prevalent in the community.

Our study shows many more locations positive for VRE than MRSA in Israeli sewage (69% and 19%, respectively (P = 0.0078) (Fig 2) but data published by the Israeli National Center for Infection Control (2013 annual summary–general hospitals) shows much higher incidences of MRSA bacteremia in Israeli general hospitals than VRE (≈20% and 5%, respectively /100,000 patients a day). The increased presence of VRE upon MRSA in Hospital and Community sewage may be due to the differences in the bacteria's preferences. MRSA is typically nasal or dermal while VRE is intestinal. There could also be differences in survival rates of the bacteria in sewage environments. Though sewage is an environment, containing water and nutrients, it may have extreme living conditions such as fluctuating levels of energy sources, oxygen, temperature, pH and osmolarity. [41]. Many polluting factors such as metals, chemicals, detergents and oils resulting from the industry may also affect bacteria survival in sewage.

We conclude that (1) our upgrade of the pour plate method for heterotrophic plate count technique using commercial selective agar plates can be a useful tool for routine screening and monitoring of the population for pan-resistant bacteria using sewage and (2) that the content of MDR bacteria in sewage represents the distribution of pan-resistant bacteria in the population and that (3) similar resistant bacteria are present in the Hospitals and in in the Community in Israel.

More research should be conducted in order to estimate the possible use of environment sampling to predict outbreaks of pan-resistant bacteria in the population and to look for other resistance genes such as *bla*_OXA-48_, *bla*_VIM_ and *bla*_IMI_ in sewage systems.
