# Correction: Population Screening Using Sewage Reveals Pan-Resistant Bacteria in Hospital and Community Samples

**DOI:** 10.1371/journal.pone.0170538

**Published:** 2017-01-12

**Authors:** The PLOS ONE Staff

The Acknowledgments section is missing from this paper. The Acknowledgments sections is: This work was performed in partial fulfillment of the requirements for the M.Sc. degree for LMG.

The following text, which appears as the fifth paragraph of the Results, should not be included in the Results, but instead in the Table 1 legend: “The State of Israel was divided into four main areas for sewage sampling; Haifa district in the north, Central district including Tel-Aviv and the Shafdan-STF, Jerusalem City in the East and the South district. Each district was divided to sub-districts. The boundaries and population size are mentioned for each sampling site. The number and percentage of positive samples for pan-resistant bacteria; CRE (blaKPC and blaNDM-1), MRSA and VRE out of the number of samplings analyzed is indicated.

All geographical coordinates were done using "Google maps" (https://www.google.co.il/maps).

# Haifa sampled twice in the same location therefore no percentage is presented.

* According to Central Bureau of Statistics (CBS) Israel 2012.

** Tel Aviv-Jaffa, Ramat-Gan, Bnei-Brak, Giv'atayim, Petah-Tikva, Giv'at-Shmuel, Kiryat-Ono, Tel-HaShomer, Ramat Ef'al, Azur, Bat-Yam, Holon, Rishon- Lezion and Rehovot”

The publisher apologizes for these errors. Please see the complete, correct Table 1 below.

**Table 1:** Sampling sites information.

Sampling Sites	Boundaries	Geographical coordinates (Latitude, Longitude)	Population*	Contains Hospital sewage	Resistance genes			
					*bla*_KPC_	*bla*_NDM-1_	MRSA	VRE
					#of positive samples/# of sampling sites			
	***Haifa District***		**462,670**					
**Haifa**	Haifa, Nesher, Krayot and Tirat HaCarmel	32.785965, 35.051865		Yes	2/2	0/2	1/2	1/2
	***Tel Aviv District and Central Region***		**1,620,838**					
**Tel Aviv (TLV)**			**414,565**					
C121 TLV	Kfar Shalem TLV			No	6/8 (75%)	0/8 (0%)	2/8 (25%)	5/8 (63%)
C114 TLV	Kfar Shalem TLV	32.045043, 34.813014		No				
C58 TLV	Yad Eliyahou	32.044491, 34.810208		Yes				
B Line TLV	North TLV	32.061493, 34.787787		No				
A23 Igudan	North TLV &Petah Tikva	32.102600, 34.781872		Yes				
C1 Igudan	South and Central TLV	32.100516, 34.800912		Yes				
Basa Igudan	South TLV & Jaffa	32.100516, 34.800912		No				
Shafdan	**	32.058599, 34.759402		Yes				
	***Jerusalem City (J-m)***	32.100893, 34.901318	**815,308**					
Sorek	West and South J-m			Yes	2/3 (67%)	1/3 (33%)	0/3 (0%)	2/3 (67%)
Og	North and East J-m	31.758565, 35.101516		Yes				
Kidron	South-East J-m	31.800825, 35.258332		No				
	***South District***	31.766390, 35.235947						
Kuseife	Kuseife		17,543	No	2/3 (67%)	1/3 (33%)	0/16 (0%)	3/3 (100%)
Be'er Sheva	Be'er Sheva	31.235874, 35.090786	197,269	Yes				
Rahat	Rahat	31.337678, 34.684747	56,943	No				
**All of Israel**		31.384765, 34.717849			**12/16 (75%)**	**2/16 (13%)**	**3/16 (19%)**	**11/16 (69%)**

The State of Israel was divided into four main areas for sewage sampling; Haifa district in the north, Central district including Tel-Aviv and the Shafdan-STF, Jerusalem City in the East and the South district. Each district was divided to sub-districts. The boundaries and population size are mentioned for each sampling site. The number and percentage of positive samples for pan-resistant bacteria; CRE (blaKPC and blaNDM-1), MRSA and VRE out of the number of samplings analyzed is indicated. All geographical coordinates were done using "Google maps" (https://www.google.co.il/maps).

^#^ Haifa sampled twice in the same location therefore no percentage is presented.

* According to Central Bureau of Statistics (CBS) Israel 2012.

** Tel Aviv-Jaffa, Ramat-Gan, Bnei-Brak, Giv'atayim, Petah-Tikva, Giv'at-Shmuel, Kiryat-Ono, Tel-HaShomer, Ramat Ef'al, Azur, Bat-Yam, Holon, Rishon- Lezion and Rehovot
